# Hong Kong Corpus of Chinese Sentence and Passage Reading

**DOI:** 10.1038/s41597-023-02813-9

**Published:** 2023-12-14

**Authors:** Yushu Wu, Chunyu Kit

**Affiliations:** Department of Linguistics and Translation, City University of Hong Kong, Hong Kong, China

**Keywords:** Interdisciplinary studies, Human behaviour

## Abstract

Recent years have witnessed a mushrooming of reading corpora that have been built by means of eye tracking. This article showcases the *Hong Kong Corpus of Chinese Sentence and Passage Reading* (HKC for brevity), featured by a natural reading of logographic scripts and unspaced words. It releases 28 eye-movement measures of 98 native speakers reading simplified Chinese in two scenarios: 300 one-line single sentences and 7 multiline passages of 5,250 and 4,967 word tokens, respectively. To verify its validity and reusability, we carried out (generalised) linear mixed-effects modelling on the capacity of visual complexity, word frequency, and reading scenario to predict eye-movement measures. The outcomes manifest significant impacts of these typical (sub)lexical factors on eye movements, replicating previous findings and giving novel ones. The HKC provides a valuable resource for exploring eye movement control; the study contrasts the different scenarios of single-sentence and passage reading in hopes of shedding new light on both the universal nature of reading and the unique characteristics of Chinese reading.

## Background & Summary

Over the past two decades, researchers have given increasing attention to reading behaviours and conducted in-depth investigations into when and where the underlying cognitive mechanisms of reading concurrently function by using recordings of physiological signals from human organs (e.g., lung, heart, eye, and brain)^1^. As one of the most prominent types of empirical data, eye movements possess unique advantages in representing accurately sliced time segments (e.g., first fixation duration, second go-past duration, and total reading time), flexibly segmented interest areas (e.g., local words and phrases or global sentences and paragraphs), and high ecological validity that allows for previewing and reviewing texts. Along with this direction, a growing number of eye-tracking datasets have been developed in recent years^2–15^ (see details in Table 1). However, it is noteworthy that the few Chinese reading corpora, such as GECO-CN^12^, BSC^13^ and CEMD^14^, were not published until last year.

**Table 1:** Introduction of eye-tracking datasets across different languages.

Corpus names (abbreviations)	Language	Participants	Word tokens read by one participant	Accumulated word tokens^1^
Dundee Corpus	English L1 & French L1	10 native speakers each	Tokens: 56,216 (types: 9,776); newspaper texts	1,083,890
			Tokens: 52,173 (types: 11,321); newspaper texts	
Potsdam Sentence Corpus (PSC)	German	222 native speakers	Tokens: 1,138; Sentences: 144	252,636
Dutch Eye-Movements ONline Internet Corpus (DEMONIC)	Dutch	55 native speakers	Tokens: 1746; Sentences: 224	96,030
Balanced Corpus of Contemporary Written Japanese (BCCWJ-EyeTrack)	Japanese	24 native speakers	Bunsetsu^2^: 411 out of 1643; 20 newspaper texts	9,864
Ghent Eye-Tracking Corpus (GECO)	Dutch L1 & English L2	19 unbalanced bilinguals	Tokens: 59,716 (types: 5,575); Gulliver’s Travels I	1,134,604
			Tokens: 54,364 (types: 5,012); Gulliver’s Travels II	1,032,916
	English	14 monolinguals	Tokens: 54,364 (types: 5,012); Gulliver’s Travels	761,096
Provo Corpus	English	84 native speakers	Tokens: 2,689 (types: 1,197); Passages: 55	145,206
Zurich Cognitive Language Processing Corpus (ZuCo)	English	12 native adults	Tokens: 21,629; Sentences: 1107	259,548
Russian Sentence Corpus (RSC)	Russian	96 Russian participants	Tokens: 1,362; Sentences: 144	196,128
Beijing Sentence Corpus (BSC)	Chinese	60 native speakers	Tokens: 1,685; Sentences: 120	101,100
Multilingual Eye-Movement Corpus (MECO)	Dutch	45 native speakers	Tokens: 2231; Sentences: 112	100,395
	English	46 native speakers	Tokens: 1540; Sentences: 112	70,840
	Estonia	52 native speakers	Tokens: 2109; Sentences: 99	109,668
	Finnish	49 native speakers	Tokens: 1487; Sentences: 110	72,863
	German	45 native speakers	Tokens: 2027; Sentences: 115	91,215
	Greek	45 native speakers	Tokens: 2083; Sentences: 99	93,735
	Hebrew	47 native speakers	Tokens: 1950; Sentences: 121	91,650
	Italian	54 native speakers	Tokens: 2114; Sentences: 90	114,156
	Korean	32 native speakers	Tokens: 1796; Sentences: 101	57,472
	Norway	42 native speakers	Tokens: 2106; Sentences: 116	88,452
	Russian	46 native speakers	Tokens: 1894; Sentences: 107	87,124
	Spanish	48 native speakers	Tokens: 2412; Sentences: 98	115,776
	Turkish	29 native speakers	Tokens: 1697; Sentences: 104	49,213
Ghent Eye-tracking COrpus of sentence reading for Chinese-English bilinguals (GECO-CN)	Chinese L1 & English L2	32 bilinguals	Tokens: 59,403 (types: 5053); Sentences: 5066	1,900,896
			The Mysterious Affair at Styles (Chapters 1–7)	
			Tokens:56,841 (types: 5363); Sentences: 5242	1,818,912
			The Mysterious Affair at Styles (Chapters 18–13)	
Copenhagen Corpus of eye tracking recordings from natural reading of Danish texts (CopCo)	Danish	22 native speakers	Tokens: 34,897; Sentences: 1,832; speech manuscripts	767,734
Chinese Eye-Movement Database (CEMD)	Simplified Chinese	1,718 native speakers	Types: 8551; Sentences: 8015	1,339,960^3^
TURead	Turkish	196 native speakers	Tokens: 2943 (types: 2185)	576,828
			192 short texts, each composed of 1–3 sentences	

*Note*. ^1^The accumulated word tokens are roughly calculated by the multiplication of tokens and the number of participants. ^2^A Japanese bunsetsu unit is composed of a content word plus functional morphology. ^3^Notice that this digit indicates the number of total fixation points but not accumulated word tokens.

The rapid growth of eye-movement corpora has boosted a variety of empirical studies that address new challenges arising from reading. In reading research with alphabetic languages, the *Dundee Corpus* promotes the discussion on word processing in parafoveal and foveal vision^2,16^, while the PSC is employed to examine word surprisal effects^3,17^, the *Provo Corpus* to investigate undersweep fixations in multiline contexts^8,18^, the GECO to explore the age-of-acquisition effect on fixations regardless of word length and frequency^6,19^ and the ZuCo to train machine learning models to predict human reading behaviours^7,20–22^. In reading research on logographic (or syllabic) languages, BCCWJ-EyeTrack is leveraged to compare clause boundary categories, showing evidence for a divergent clause wrap-up effect from those in alphabetic scripts^5,23,24^. BSC is utilised to capture the interplay effects of complexity and predictability on the *preferred viewing location* (PVL) in Chinese reading, indicating that fixations tend to locate closer to word centres for words with lower visual complexity and higher predictability^13,25^.

Nevertheless, many key issues remain unaddressed in Chinese reading. First, Chinese passage reading remains largely understudied, leaving a sharp contrast to the existing studies of passage reading in alphabetic languages. The material types in the largest Chinese reading corpora, BSC and CEMD, are tailored only to unrelated sentences and hence do not aim to reveal across-sentence reading effects or behavioural differences between readings of unrelated and coherent sentences. Second, there has not been any natural reading corpus that records both sentence and passage reading for a typical logographic language such as Chinese. Third, CEMD plainly amalgamates the sentence reading data from 57 controlled experiments into one large collection, disregarding variations arising from many subjects and items. In this context, it is particularly significant to develop HKC as a large-scale eye-tracking corpus of natural reading of Chinese sentences and passages. Different from other reading corpora, it provides valuable data not only for in-depth examinations of typical characteristics of natural reading but also for comparison of eye-movement patterns in reading unrelated and coherent sentences. In addition to the reusability and versatility of its data for exploring topics in Chinese reading, HKC also has untapped potential to grow by expanding the pool of texts and subjects for a wider scope of reading research through the unique cultural environment of a trilingual society in Hong Kong. Specifically, we can extend its current version, which records native Mandarin speakers, to native Cantonese speakers reading traditional Chinese and even bilingual/trilingual individuals reading simplified and traditional Chinese and English.

**Fig. 1:**
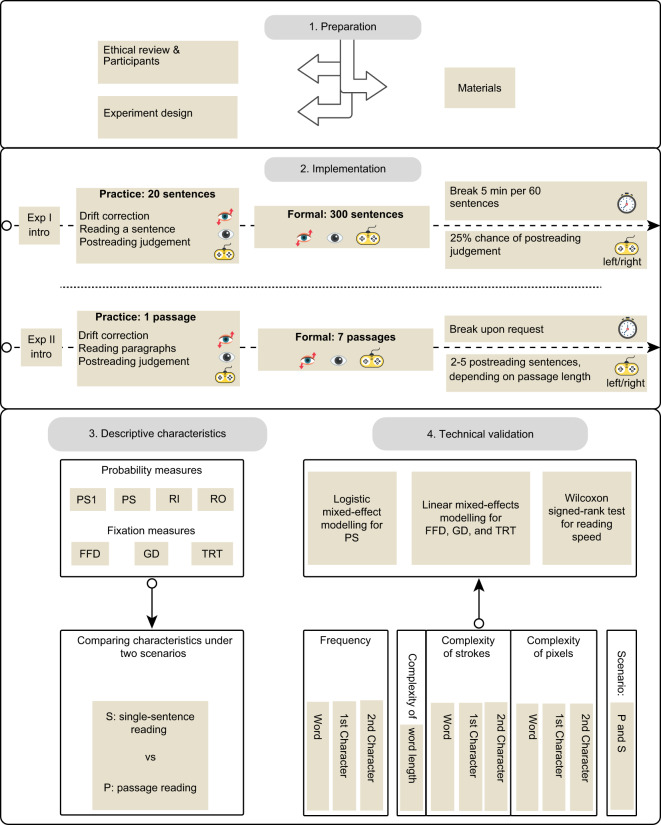
Schematic overview of HKC development.

Here, we present HKC, a large-scale eye-tracking corpus that records 98 young adults reading 300 single sentences and seven passages in simplified Chinese and underscores the importance of considering natural reading. For an easy grasp of how HKC is developed, Fig. 1 illustrates a schematic overview of its preparation, implementation, descriptive characteristics, and validation. The development of HKC is intended to serve a wide range of studies, among which two are particularly worth mentioning and hence showcased in this article. One is to validate whether the previously revealed effects of word frequency and complexity^9,14,26^ can be replicated in natural reading, and the other is to investigate potential differences in reading performance (e.g., reading speeds, skipping probability, and fixation durations) between two contrasting scenarios: single-sentence versus passage reading.

## Methods

### Participants

This study was approved in advance of implementation by the Human Subjects Ethics Subcommittee of the corresponding college. We recruited 98 university students (89 females, age = 26 ± 3.64) as our test-takers, who are native speakers of Mandarin, skilled in reading simplified Chinese with normal (or corrected-to-normal) eyesight and no illness that impacts cognitive abilities. They each signed a consent form before the experiment and received monetary remuneration upon completion. Due to privacy protection, other information is not disclosed.

### Apparatus

Two experiments take advantage of the following hardware: (1) tower-mounted EyeLink 1000 series (SR Research, Canada) with a sampling rate up to 1000 Hz and a spatial resolution of 0.01° of visual angle; (2) an 18-inch ViewSonic CRT monitor (resolution rate, 1024 × 768 pixels; and refresh rate, 85 Hz); and (3) an adjustable chin rest.

### Materials

The top 300 sentences that were 30 characters long (including punctuation marks) were selected from the XIN subcorpus of the Chinese Gigaword Corpus^27,28^ by first sorting sentences in ascending order of average entropy per character (i.e., the overall information per language signal^29^). Entropy is estimated by a simple unigram model using character frequencies in the corpus, followed by filtering out those entries suspected of any lexical, syntactic or semantic inclinations (e.g., long numbers of many digits and repeated expressions) or other bias (e.g., religion, racism, sexuality, and violence). We opted to choose the most likely unbiased sentences in this way in hopes of smoothing the natural reading process to the greatest extent possible. The sentence length was chosen to allow a full utility of the screen width, except for a necessary margin on four sides (left and right, 110 pixels; top and bottom, 180 pixels).

Following a similar procedure with the same criteria except for text length, 7 passages were selected from the same corpus with no overlap with any selected single-lined sentences (see Table 2 for sample materials). Totalling a text length of 8742 characters, these passages cover a variety of topics, including 1 on celebrity news, 1 on city development, 2 on education, 1 on employment, and 2 on sports. A small number of uncommon words, such as technical terms and long numbers, were pruned out or replaced with easier or shorter ones without altering the meanings of original sentences. For the best fit to the monitor, we divided each passage into a title page and several content pages (individually: 6, 4, 5, 6, 5, 10, and 5 for the seven passages, in a total of 41) according to the text length (in number of characters), and configured each page with 9 lines (unless the last page of a passage) and each line with 38 characters (unless the last line of a paragraph). There are 1078 ± 275 characters per passage, 36* ± *21 sentences per passage, and 40 ± 21 characters per sentence.

**Table 2:** Samples of single sentence and passage paragraph.

Materials	Chinese texts	Pronunciation (in Chinese Pinyin)	English Translation
Single sentence	日本要想在会上与西方国家保持协调, 必须得到美国的支持和理解。	Rìběn yàoxiǎng zài huì shàng yǔ xīfāng guójiā bǎochí xiétiáo, bìxū dédào Měiguó de zhīchí hé lǐjiě.	If Japan wants to continue to coordinate with the Western countries on the meeting, it must get the US’s support and understanding.
	江苏队的核心队员是国手胡卫东, 全队的战术是以他为中心制定的。	Jiāngsū duì de héxīn duìyuán shì guóshǒu Hú Wèidōng, quán duì de zhànshù shì yǐ tā wéi zhōngxīn zhìdìng de.	The centerpiece of the Jiangsu team was the national player Hu Weidong and the team’s tactics centre on him.
	有超过半数的在港德国公司表示, 会继续扩大其在亚洲地区的业务。	Yǒu chāoguò bànshù de zài Gǎng Déguó gōngsī biǎoshì, huì jìxù kuòdà qí zài Yàzhōu dìqū de yèwù.	More than half of German companies in Hong Kong said they would continue to expand their business in the Asian region.
Passage paragraph	其中, 遭受批评最严重的, 是高考千军万马过独木桥的剧烈竞争给孩子身心带来巨大压力。据了解, 虽然国家近年来每年都扩招20%以上, 但是, 高校在校学生不到同龄人总数的7%。“我觉得他们太累了”。王少华的女儿今年上高二, 已经在日以继夜地准备高考。她每天有做不完的作业, 考不完的试。大部分日子都是早晨6点起床, 晚上24时左右睡觉。	Qízhōng, zāoshòu pīpíng zuì yánzhòng de, shì gāokǎo qiānjūn-wànmǎ guò dúmùqiáo de jùliè jìngzhēng gěi háizi shēnxīn dàilái jùdà yālì. Jù liǎojiě, suīrán guójiā jìnnián lái měi nián dōu kuò-zhāo 20% yǐshàng, dànshì, gāoxiào zài-xiào xuéshēng bù dào tónglíngrén zǒngshù de 7%. “Wǒ juédé tāmen tài lèi le”. Wáng Shàohuá de nǚér jīnnián shàng gāo-èr, yǐjīng zài rìyǐjìyè de zhǔnbèi agāokǎo. Tā měi tiān yǒu zuò bù wán de zuòyè, kǎo bù wán de shì. Dà bùfèn rìzi dōu shì zǎochén 6 diǎn qǐchuáng, wǎnshang 24 shí zuǒyòu shuìjiào.	Among these factors, the most criticised one is the fierce competition of Gaokao (college entrance examination), like hordes of troops and horses crossing a single-plank bridge, which causes huge physical and mental stress on the kids. As far as we know, although the country has expanded its annual university intake by more than 20% in recent years, university students still account for less than 7% of their peers. “I think they are too tired”, uttered Wang Shaohua, whose daughter is in the second year of high school this year but has already begun preparing for Gaokao around the clock. She has countless homework and tests every day. Most days she gets up at 6 a.m. and goes to bed around 24 p.m.

**Fig. 2:**
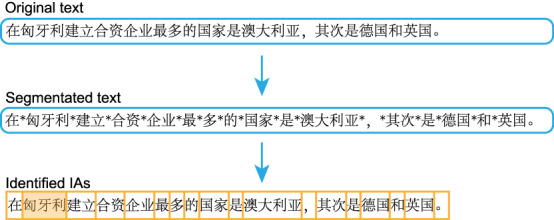
Procedure of word segmentation and sample of segmentation results in materials. *Note*. Pinyin: Zài Xiōngyálì jiànlì hézī qǐyè zuìduō de guójiā shì Àodàlìyà, qícì shì Déguó hé Yīngguó. Meaning: The country that has established the most joint ventures in Hungary is Australia, followed by Germany and the United Kingdom.

Given the convention of no interword spacing in Chinese texts, we performed word segmentation following the national standard GB/T 13715-92 (1992) and inserted delimiters (*) between words in the materials for the purpose of facilitating word-based analyses after data collection (see Fig. 2). We manually checked the results of the segmentation word by word and resolved ambiguous and controversial cases according to our best understanding of the standard. Experiment Builder software (SR Research, Canada) automatically specifies an interest area (IA) between two delimiters and keeps all delimiters invisible to participants during reading. Each punctuation mark was also taken as an IA.

Taken together, the materials contain 10,117 word tokens (of 2,160 types) with a mean word stroke of 11 (* ± *6) congruent with the previous metrics^30^. Single sentences include 5,150 word tokens (of 1354 types) and passages 4,967 ones (of 1,434 types). We extracted information on word frequency from the Chinese Lexical Database (CLD)^29^. The distributions of word tokens over lengths (of 1, 2, 3, and 4 characters) are 46.3%, 50.4%, 3.1% and 0.2%, respectively, and over frequency (of low, medium, and high) are 43.4%, 22.4% and 34.2%, respectively. Both the single sentence and passage subsets exhibit similar distributions over the word frequency and length (see Table 3 for more details).

**Table 3:** Word-length and word-frequency occurrences in the materials.

Word information		Number of word tokens in the materials		
Word numbers		Overall	Single sentences	Passages
		10,117	5,150	4,967
Word length	1	4,683 (46.3%)^1^	2,379 (46.2%)	2,304 (46.4%)
	2	5,102 (50.4%)	2,658 (51.6%)	2,444 (49.2%)
	3	312 (3.1%)	108 (2.1%)	204 (4.1%)
	4	20 (0.2%)	5 (0.1%)	15 (0.3%)
Frequency^2^	Low	4,392 (43.4%)	2,271 (44.1%)	2,121 (42.7%)
	Medium	2,269 (22.4%)	1,097 (21.3%)	1,172 (23.6%)
	High	3,456 (34.2%)	1,782 (34.6%)	1,674 (33.7%)

*Note*. 1 n (%). 2 Low: less than 100; Medium: 100 to 1000; and High: greater than 1000 ipm (instances per million).

### Experimental design

There are two varieties of experimental designs for collecting eye-movement data in reading: factorial and nonfactorial designs. The former tests two or more independent variables (IVs) driven by well-defined hypotheses, while the latter controls only one IV and entails exploring data (or effects on reading performance in a way yet to be clarified) and generating hypotheses. The two types of design differ in the rigidity of manipulating variables but complement each other in terms of methodology. Given our goal of observing the scenario effect and exploring more probable patterns in natural reading, we adopted a nonfactorial design and controlled no other variable except the reading scenarios (to be either single sentences or passage reading).

Regarding the two scenarios, we created two within-subject experiments respectively (Exp I for sentence reading and Exp II for passage reading) with Experiment Builder (SR Research, Canada). The design of each experiment involves considerable preparation for five main parts for the designer to carry out: eye-tracking configurations, experiment instructions, text materials for formal reading, post-reading comprehension questions, and experiment implementation with special settings for real-time message collection (e.g., button pressing for page flipping in natural reading). There were pilot runs for the latter, which was intended to record all real-time happenings on the participant computer, including those that indicate the time points of page flipping at the end/beginning of reading the current/next page. Such data were used later for delimiting reading period (see details in the *Data preprocessing*).

### Experimental procedure

The experimental procedure involves three reading tasks for subjects (Ss) to perform: warm-up reading (T1), formal reading (T2), and post-reading comprehension (T3), for the purpose of collecting both behavioural and eye-movement data. The former type of data is collected from Ss pressing buttons to flip pages during T2 and to answer questions for T3, and the latter type from Ss’ dextral eyes during their reading in T2. All Ss successively completed all T1, T2, and T3 of both Exp I and Exp II in a windowless silent booth under identical conditions. Half of the Ss first performed Exp I followed by Exp II, while the other half followed a similar procedure to perform Exp II ahead of Exp I. It took approximately one hour to finish the two experiments with a rest of 5–10 minutes in between. All Ss successfully completed two experiments.

The optimal typography was piloted in advance. We configured single sentences in 18-pt Simsun font, horizontally left-aligned and vertically centred-aligned, with a monitor-to-subject distance of 64 cm; passages were in 16-pt Simsun font, horizontally justified alignment, and double-line spacing, with a monitor-to-subject distance of 55 cm. Notwithstanding the two typography settings, we formatted each Chinese character to extend at 0.85° of visual angle uniformly for both reading scenarios. According to our test runs on typographic settings, passage texts in the smaller font and double spacing could lead to fewer return sweeps, fewer fixations close to screen corners, and less undesired crossline interference.

#### Warm-up reading (T1)

Before formal reading, we instructed the participants to read silently at their own pace and familiarise themselves with the experimental procedure through two practice sessions, as shown in the *Implementation* in Fig. 1. The length and content of the practice sentences differ from those in formal reading to prevent developing a practice effect.

#### Formal reading (T2)

**Fig. 3:**
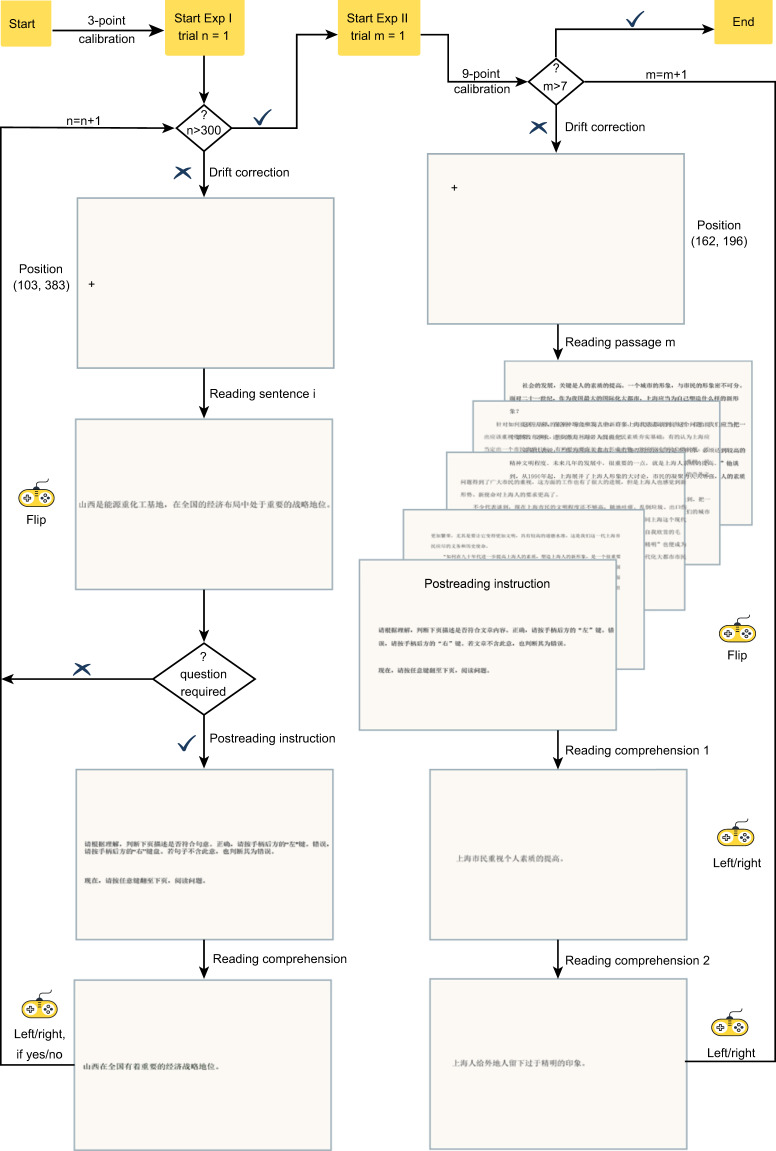
Reading procedure for 300 sentences and 7 passages.

The procedure of formal reading is similar to that of warm-up reading. Figure 3 exhibits a sample procedure of a subject reading sentences in Exp I and passages in Exp II. In Exp I, Ss had a 3-point calibration and drift correction before one-line sentence reading. Ss had a five-minute break after reading every 60 sentences to prevent a fatigue effect. In Exp II, Ss first had a 9-point calibration and drift correction before beginning multipage passage reading. To sustain their regular reading performance, Ss could rest after reading an entire passage. Additionally, we randomised the order (i.e., trial index) of materials presented to readers to prevent an order effect.

#### Post-reading comprehension task (T3)

The post-reading tasks required the Ss to provide a Yes/No answer (by pressing the left/right button of the response handle) to whether the meaning of a post-reading sentence (referred to as a comprehension question in order to avoid confusion with text sentences for formal reading) matched the content of formal reading, for evaluating their grasp of the overall message and reminding them to concentrate on reading. In Exp I, we manually formulated 75 comprehension questions for 75 designated sentences, so Ss should answer one of them after reading four sentences on average. The probability of 25% (75 out of 300 sentences) is to ensure a reading task without too much comprehension load^31^ because our goal is to capture natural reading, rather than hard-working reading that happens only in labs but not in real life. For Exp II, we formulated 25 comprehension questions and assigned them to passages in a way that longer ones are assigned more, in the range of 2 to 5 questions per passage (individually: 4, 2, 3, 4, 4, 5, and 3, in a total of 25).

### Data preprocessing

Using Eyelink Data Viewer (DV, SR Research, Canada), we first screened the behavioural data, which show that Ss pressed buttons as required without exception. Then, we calculated the accuracy rates of subjects’ button responses (1: correct; 0: incorrect; −1: no response required) for both experiments. Average rates of 85% and 88% accuracy were obtained for sentence and passage reading, respectively, indicating an overall quality level of reading between those of the RSC (80%)^9^ and BSC (90%)^13^. Regardless of accuracy rates, all subject eye-movement data are included in HKC because our main task is to collect real data from natural reading that is supposed to allow for various levels of reading comprehension.

Using DV, data retrieval for Exp I is straightforward, given the setting of one single-line sentence per full-screen page. However, a technical problem arises from data retrieval for Exp II: eye-tracking data for reading content pages were overlaid densely on the title page. To resolve this problem, we used the messages of subjects’ button pressing for page flipping to delimit the entire reading period of a passage into subordinate interest periods (IPs) that identified their page numbers. This method facilitates our data analysis despite missing eye-movement data for the time lags between button pressing and page flipping; these periods were too short (less than 10 milliseconds) to indicate any significant language processing and hence can be disregarded with no harm. Subsequently, we excluded the data from title pages, which reflect the reading performance of single sentences rather than paragraphs. Finally, the data resulting from Exp I and Exp II are stored in two files (UTF-8 encoded): a sentence subcorpus and a passage subcorpus, which jointly form the current version of HKC^32^.

The eye-tracking data collected with a 1000 Hz sampling rate was aggregated per IA. We excluded the eye-tracking data related to punctuation and blinks (when no pupils were detected), marked the data without fixations as NA, and filtered out single fixations shorter than 80 ms or longer than 1000 ms (for the reason of not revealing linguistic processing^14,33^). As a result, 3.1% of the total eye movement data was excluded. A total of 980,326 out of 1,149,411 data points are valid, and the number shows that HKC is a sizeable reading corpus among the existing representative ones (see Table 1 for a comparison), although not the largest.

## Data Records

HKC is released on the *Open Science Framework* (OSF) repository^32^ under the licence of CC BY 4.0 for free access via the identifier doi 10.17605/OSF.IO/Z465B. A set of files are presented in this repository, including (1) datasets (“sentence subcorpus.rda”, “passage subcorpus.rda”, and “HKC.rda”), (2) materials (“materials.xlsx” and “punctuation_distribution.xlsx “), (3) variable definitions (“definitions.csv”), and (4) accuracy information (“accuracy sentence.xlsx” and “accuracy passage.xlsx”).

The files “sentence subcorpus.rda” and “passage subcorpus.rda” store eye-movement measures of reading unrelated and contextually coherent sentences, respectively, and the file “HKC.rda” provides a unified eye-movement dataset of the two, which is fit for direct data loading in an R language environment. Users can transfer it to any other format of their interest by converting it to a data frame and then writing it to other formats. In this released version of HKC, each IA is in a row consisting of a list of eye-movement measures whose definitions are presented in Table 4. The file “materials.xlsx” contains two sheets of the materials, consisting of 300 sentences and 7 passages, with words segmented by delimiters “*” and answers to decision tasks attached. The file “punctuation distribution.xlsx” summarises the distribution of 11 punctuation marks across the two types of materials, and the file “definitions.csv” summarises the variables used in HKC. Two more sheets, “accuracy sentence.xlsx” and “accuracy passage.xlsx”, provide participants’ actual button response (6: left and 7: right) and the corresponding accuracy (1: correct, 0: incorrect, and −1: no button response required) in Exp I and II, respectively.

**Table 4:** The variables used in the released version of HKC.

No.	Variable names (abbreviations)	Definitions
1	FORMAT	The source of materials (two-level, S: single sentences vs. P: passage).
2	RECORDING_SESSION_LABEL	The order of participants.
3	SENTENCE_OR_PASSAGE_NUMBER	Unique number for materials (S: 1 to 300; P: 1 to 7).
4	TRIAL_INDEX	The sequential trial order of the real-time recording (S: 1 to 300; P: 1 to 7).
5	IP_INDEX	Page number in a passage (the number is set to 1 for 300 single sentences).
6	IA_ID	The ordinal number of the current word (as an interest area) per page.
7	WORD	The visual form of each Chinese word.
8	TRIAL_DWELL_TIME	Summation of all fixation durations for the whole trial.
9	TRIAL_FIXATION_COUNT	Total number of fixations in the whole trial.
10	TRIAL_TOTAL_VISITED_IA_COUNT	Total number of unique interest areas visited over the whole trial.
11	IA_AVERAGE_FIX_PUPIL_SIZE	Average pupil size across all fixations.
12	IA_DWELL_TIME^1^	Total reading time, or dwell time (i.e., summation of the durations across all fixations) on the current interest area.
13	IA_DWELL_TIME_PER	Percentage of trial time spent on the current interest area.
14	IA_FIRST_FIXATION_DURATION (FFD)	Duration of the first fixation event within the current interest area regardless of fixation counts.
15	IA_FIRST_FIXATION_X	The X position of the first fixation event within the current interest area.
16	IA_FIRST_FIXATION_Y	The Y position of the first fixation event within the current interest area.
17	IA_FIRST_RUN_DWELL_TIME^2^	The total duration of all fixations in the first run of fixations on the current interest area.
18	IA_FIRST_RUN_FIXATION_PER	Percentage of all fixations in a trial falling in the first run of the current interest area.
19	IA_FIRST_RUN_FIXATION_COUNT	Number of all fixations in a trial falling in the first run of the current interest area.
20	IA_FIRST_RUN_LAUNCH_SITE	Pixels of the horizontal position of the fixation immediately preceding the current interest area, to the left edge of the interest area.
21	IA_FIXATION_PER	Percentage of all fixations in a trial falling in the current interest area
22	IA_FIXATION_COUNT	Total number of fixations falling in the interest area
23	IA_REGRESSION_IN (RI)	Whether the current interest area received at least one regression from later parts of the sentence. 1 if yes; 0 if not.
24	IA_REGRESSION_OUT (RO)	Whether a regression(s) was made from the current interest area to earlier parts of the sentence prior to leaving that interest area in a forward direction. 1, if yes; 0 if not.
25	IA_SELECTIVE_REGRESSION_PATH_DURATION	Total fixation duration starting from eyes first fixation within the current interest until the eyes enter an interest area of a higher ID.
26	IA_SKIP (PS1)	An interest area is considered skipped (i.e., IA_SKIP = 1) if no fixation occurred in the first go-past time.
27	IA_SKIP_FULL (PS)	The probabilities of an IA’s fixation count being “0”, specifying that this area was not fixated on during the whole process of sentence reading.
28	IA_SPILLOVER	The duration of the first fixation made on ‘interest area (n + 1)’ after leaving the current ‘interest area n’ in the first-pass time.

*Note*. ^1^Also known as total reading time (TRT). ^2^Also known as gaze duration (GD).

Table 5 exhibits an array of unique descriptive characteristics of HKC in terms of a series of key measures which provide prominent contrasts between the two reading scenarios (S: single-sentence reading and P: passage reading). Specifically, P has higher skipping rates (as reflected in probabilities of the PS1 and PS), lower regression rates (in the probabilities of RI and RO), and shorter FFD, GD, and TRT (despite their large SDs) than S. All of these agree nicely with native speakers’ language intuition about the contrast of the two scenarios. In particular, it is hypothesized that richer contextual information establishes better coherence and continuity of reading and gives a strong account for the better reading performance in the passage reading scenario.

**Table 5:** Descriptive characteristics in HKC.

Eye-movement measures	HKC (N = 980,326)	Single sentences (N = 504,594)	Passages (N = 475,732)
PS1	659,722 (67%)^1^	288,583 (57%)	371,139 (78%)
PS	485,714 (50%)	198,937 (39%)	289,777 (61%)
RI	170,902 (35%)	113,424 (37%)	57,478 (31%)
RO	100,309 (20%)	66,958 (22%)	33,351 (18%)
FFD	228.11 (94.77)^2^	231.28 (95.75)	219.16 (91.37)
GD	240.09 (123.29)	247.86 (130.72)	227.23 (108.68)
TRT	351.59 (265.92)	386.44 (294.47)	293.07 (195.96)

*Note*. ^1^n (%). ^2^Mean (SD).

## Technical Validation

In addition to the above manifestation of contrastive characteristics of sentence and passage reading, the effects of word frequency, visual complexity, and reading scenario on eye-movement measures in HKC provide further validation, into which we delved by (generalised) linear mixed-effects models ((G)LMMs) and the Wilcoxon signed-rank test. Since HKC does not include any annotation of lexical properties (e.g., length, stroke, and frequency) and is inherently a collection of eye-movement measures for individual word tokens, we resorted to CLD^29^ for a wide range of (sub)lexical properties (e.g., frequency, complexity, phoneme, and entropy, etc.). CLD offers high explanatory power in that the average deviance explained (ADE) tests indicate a higher value of CLD than those of other datasets (e.g., Chinese Gigaword^34^, SUBTLEX-CH^35^, and Leiden Weibo Corpus^36^). By Java programming, we annotated each word token of HKC with its complete list of lexical properties from CLD by using word matching to align corresponding records of the two datasets. The resulting dataset is then leveraged for our data validation using R language^37,38^.

### (G)LMMs and Wilcoxon signed-rank test

To validate the HKC, we separately constructed four (G)LMMs for four dependent variables, namely, PS, FFD, GD, and TRT (see Table 4 for definitions). The scenario is an independent variable and lexical properties below (with respective abbreviations in parentheses) covariates in our study. We treated all of them as fixed effects in the (G)LMMs:

- Frequency: frequencies of word (Frequency), 1st character (C1Frequency), 2nd character (C2Frequency), 3rd character (C3Frequency), and 4th character (C4Frequency).
- Complexity: word length (Length); number of strokes per word (Strokes), 1st character (C1Strokes), 2nd character (C2Strokes), 3rd character (C3Strokes), and 4th character (C4Strokes); and number of pixels per word (Pixels), 1st character (C1Pixels), 2nd character (C2Pixels), 3rd character (C3Pixels), and 4th character (C4Pixels).
- Scenario

Considering the repeated measures design of our experiment with Ss reading identical materials, we included random error terms as (1) an intercept for the subjects, (2) an intercept for the items, (3) a slope for scenarios across subjects, and (4) a slope for scenarios across items as four random effects in the (G)LMMs. As preliminary processing prior to fitting the (G)LMMs, all complexity measures were scaled by centring, and frequencies were converted to their logarithmic values using base 10 for the correction of the original Zipfian distributions^39^. To address the data noted as NA, the *maximum likelihood estimation* approach was applied, and parameters in the (G)LMMs were updated based on the imputed values by the expectation-maximisation algorithm. Regarding the model construction, we first fit each (G)LMM by including all the random effects (without any fixed effects). Second, we deducted the random effects one by one each time and weighed the entropy-based Akaike information criterion (manifested as AIC in R) of the updated model in the hope of settling the model with the lowest AIC. Due to the problematic convergence of random slopes, we used fixed slopes with random intercepts across items and subjects. In this way, a random-effects-ready model was selected. Third, we expanded the random-effects-ready model by adding all fixed effects at once. A backwards stepwise selection was then carried out, and we detected the noncontributive fixed effects or those with unacceptable variance inflation factors (≥5) in case of the presence of collinearity. Due to data sparsity (3.3% of the total), we deleted the sublexical properties of the third and fourth characters (C3Strokes, C4Strokes, C3Pixels, C4Pixels, C3Frequency, C4Frequency) because these properties explain very few data points and the deletion makes little difference. Finally, we built the fittest model for each (G)LMM.

**Fig. 4:**
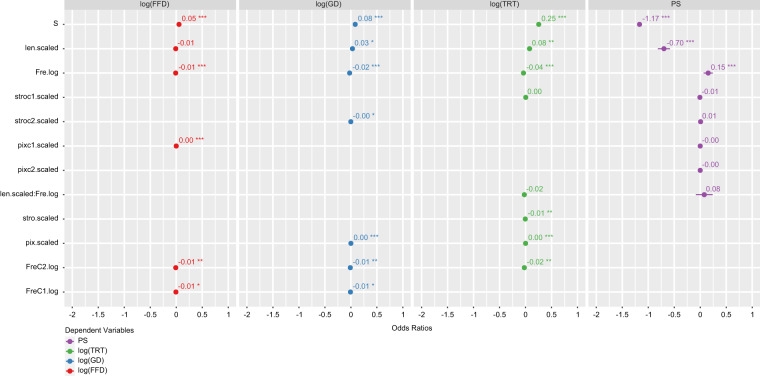
Estimates (odds ratios) of the effects given by the final (G)LMMs.

**Fig. 5:**
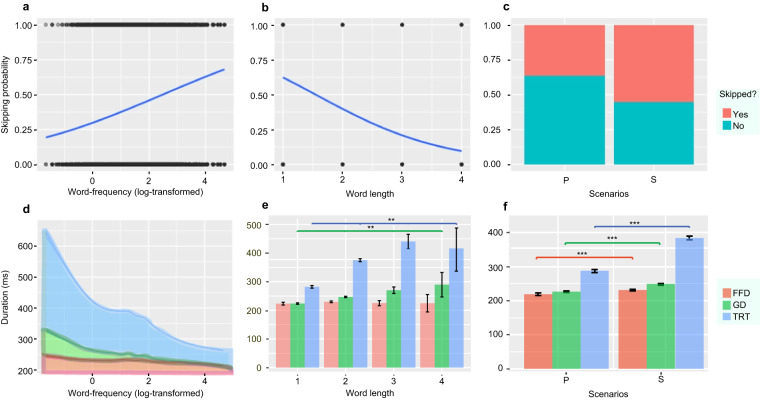
Main effects of word length, word frequency and scenario on PS, FFD, GD, and TRT. *Note*. Panels (**a**–**c**) present skipping probability in percentage, while panels (**d**–**f**) present reading time in milliseconds. Panels (**e,f**) show the mean FFD, GD, and TRT (with error bars) across word length and scenarios. *p < 0.05, **p < 0.01, ***p < 0.001.

The results from the final (G)LMMs are summarised in Table 6 and visualised in Fig. 4, suggesting that many established lexical effects in controlled experiments can also be revealed in natural reading and that sublexical factors also modulate natural reading. Three main effects on reading stand out from others, namely, word frequency, word length, and scenario. These are visualised in Fig. 5, which illustrates the effects on PS (Fig. 5a–c) and on FFD, GD, and TRT (Fig. 5d–f).

**Table 6:** Summary of the fittest (generalised) linear mixed-effects models.

Models	log(FFD)		log(GD)		log(TRT)		PS	
*Predictors*	*Estimates*	*std. Error*	*Estimates*	*std. Error*	*Estimates*	*std. Error*	*Odds Ratios*	*std. Error*
(Intercept)	5.38***	0.02	5.39***	0.02	5.58***	0.03	1.05	0.07
Fre.log	−0.01***	0	−0.01*	0.01	−0.04***	0.01	1.17***	0.05
FreC2.log	−0.01**	0	−0.01**	0	−0.02**	0.01	−3	—
FreC1.log	−0.01*	0	−0.01*	0	—	—	—	—
len.scaled	−0.01	0.01	0.04**	0.01	0.08**	0.03	0.50***	0.03
stro.scaled	—	—	—	—	−0.01**	0	—	—
stroc2.scaled	—	—	0	0	—	—	1.01	0.01
stroc1.scaled	—	—	—	—	0	0	0.99	0.01
pix.scaled	—	—	0.00***	0	0.00***	0	—	—
pixc1.scaled	0.00***	0	—	—	—	—	1	0
pixc2.scaled	—	—	—	—	—	—	1	0
len scaled * Fre.log	—	—	−0.02*	0.01	−0.02	0.02	1.08	0.09
Scenarios [S]	0.05***	0	0.08***	0	0.25***	0	0.31***	0
**Random Effects**								
σ^2^	0.13		0.17		0.32		3.29	
τ_00_	0.00_Item_		0.01_Item_		0.03_Item_		0.44_Item_	
	0.01_Subject_		0.01_Subject_		0.03_Subject_		0.23_Subject_	
ICC	0.08		0.1		0.15		0.17	
N	98_Subject_		98_Subject_		98_Subject_		98_Subject_	
	1734_Item_		1739_Item_		1739_Item_		1739_Item_	
Observations	127557		315245		318655		526515	
Marginal	0.006		0.016		0.051		0.093	
Conditional R^2^	0.084		0.114		0.192		0.246	

*Note*. *p < 0.05, **p < 0.01, and ***p < 0.001. “—” stands for a missing variable included in the corresponding final models.

Following the practice of ZuCo^7^, we conducted a paired one-tailed Wilcoxon signed-rank test to compare the average reading speeds of each participant (across all trials) under two reading scenarios by the unit of words per minute (WPM).

### Effects of word frequency

Word frequency plays a crucial role in the validation of eye-movement corpora, as shown in the studies by Laurinavichyute *et al*.^9^ and by Zhang *et al*.^14^ The effects of word frequency revealed by HKC data are presented in Fig. 5a,d, revealing that high-frequency words tend to be more efficiently processed, according to their greater skipping rates and shorter fixations, than low-frequency words. These results are evidently consistent with the results in the literature.

### Effects of visual complexity

We present the effects of visual complexity from two perspectives: word length and spatial density. Regarding the effects of word length, we found its impact on eye-tracking measures of PS, FFD (marginally significant), GD, and TRT (see Fig. 5b,e). The effect of word length on PS reveals such a trend that longer words are less likely to be skipped, which is consistent with native speakers’ intuition. Its impacts on GD and TRT show that dwell times are longer for 2-character words than for 1-character words. The rise of the reading times from 1- to 2-character words is particularly worth noting, given that the latter account for 96.7% of all words. Surprisingly, 3- and 4-character words do not necessarily demand greater cognitive effort than shorter words do, in the sense that shorter FFD, GD, and TRT characterise their reading. Intuitively, one may attribute this to the relatively efficient processing of fixed expressions and strong collocations such as idioms (e.g., *Multi-Constituent Unit Hypothesis*^40^). However, further effort is still needed to examine whether eye-movement data on such a small proportion of 3- and 4-character words (approximately 3% of the total in our data) would lend any convincing support to a conclusion such as ours.

Regarding the effects of spatial density, we uncover the lexical modulation of word strokes on TRT, that of word pixels on TRT and GD, and the sublexical effect of 1^st^-character pixels on FFD. Strokes play a crucial role in measuring the visual complexity of written words in logographic languages such as Chinese, Japanese, and Korean (CJK family), unlike alphabetic languages in which written words are measured by length in number of letters. This difference can be illustrated by contrasting two one-character words in Chinese, e.g., 水 (shui3, “water”) and 美 (mei3, “beauty”), which are of 4 and 9 strokes, respectively, giving a sharp contrast in visual complexity despite the same word length. Compared with strokes, pixels manifest visual complexity in a more delicate (or sensitive) way in that words in HKC contain greater variability in pixels (5212 ± 2148) than in strokes (11 ± 6). This can account for our findings on the sublexical effect of pixels but not strokes.

Generally, word length maintains its significant modulation across all these eye-movement measures in (G)LMMs, i.e., PS, FFD (marginally significant), GD, and TRT, suggesting that Chinese reading performance is affected more significantly by horizontal complexity than by spatial density, clearly in line with the reading of alphabetic scripts. All the above together manifest that complexity factors at both lexical and sublexical levels influence eye movements in the natural reading of Chinese texts, although more details about how they work have yet to be further explored using the available HKC data. Our results replicated the key effects of visual complexity on eye movements in reading. A longer length and a greater stroke count or pixel count tend to give rise to lower likelihoods of skipping and longer fixation durations^9,22,26,41,42^.

### Effects of the scenario

From the results yielded from the (G)LMMs (Table 6), we observed a significant modulation of reading scenario on the probability of word skipping (Fig. 5c) and on the measures of FFD, GD, and TRT (Fig. 5f). Specifically, P manifests shorter duration and greater skipping rates than single-sentence reading in S. This contrast provides evidence for an intuitive observation that among the two scenarios, the one (P) that provides richer contextual information allows more efficient reading performance than the other (S). Our findings on the scenario effect clearly justify the need for further research in this direction.

The Wilcoxon rank-sum test we conducted on HKC revealed that our participants’ reading of the passages was significantly faster than that of the single sentences (Z = 1.88, *p* < 0.001). Their average reading speeds show that Chinese readers are currently capable of reading 304 ± 182 words per minute (WPM) in S and 527 ± 277 WPM in P (with punctuation and other outliers excluded). The latter appears to have been sped up by approximately 36% from an average of 386 WPM^43^ when measured twenty-some years ago, suggesting that this generation of readers may read faster on a computer screen.

Taken together, the results we obtained from HKC about the impacts of word frequency, visual complexity of words, and reading scenarios offer dependable justification for its validity and reliability. These results not only echo the previous findings but also provide strong evidence for the usability of HKC as a large-scale dataset to facilitate exploratory linguistic and cognitive studies of Chinese reading, especially those involving multidimensional analysis (of a large number of correlated variables).

## Usage Notes

HKC is distinguished as the first Chinese reading corpus that records natural reading data in the two contrastive scenarios for sentence and passage reading. Its within-subject design, as another distinctive feature, may help bypass the data variability issue in between-subject designs for comparative research. It boosts the salience of studies of peculiar issues in Chinese passage reading, such as return-sweeps^18^ and wrap-up effects^44^, which play a significant role in the reading process but have remained severely understudied. From a broader perspective, HKC, as a valuable empirical dataset, can be used to facilitate a variety of research on Chinese reading that can deepen our understanding of eye-movement controls in logographic language reading, especially how reading scenarios and contextual factors affect where readers move their eyes next (fixation location) and when (fixation duration)^45^. It can also be leveraged as training data for machine learning to predict reading behaviours, such as how readers select a saccade landing site, how they perform word segmentation, and where they encounter reading difficulties.

The HKC is now open to the public for academic, pedagogical, or any noncommercial use. Additional measures, not released in this version, are also available upon request. Users may integrate HKC with other linguistic data in a similar fashion as we used CLD, as long as the two sets of data can be properly aligned, especially by word matching. In the R language environment, users may consider subsetting HKC data in a way that best fits their interests with the aid of the *filter* function if the large size of the original data is their concern. In addition, a number of main packages, such as dplyr^46^, ggplot2^47^, gtsummary^48^, lm4^49,50^, performance^51^, sjplot^52^, and tidyverse^53^, are recommended for summarising and normalising data and for fitting (G)LMMs.

## Data Availability

Two R scripts (“preprocessing.R” and “lmeModelling.R”), resulting from the step-by-step coding for our data preprocessing and technical validation, respectively, are released in the repository of OSF^32^. Also released is the source code file (mergeChineseInfo.java) of a Java program for integrating lexical property information of CLD for the words in HKC by means of word matching, on the premise of a standardised format (word-based and UTF-8 comma-delimited data format).
